# 5G approach: Enhancing collaboration between primary care and specialist physicians

**DOI:** 10.1002/jgf2.70049

**Published:** 2025-07-15

**Authors:** Kosuke Ishizuka, Taiju Miyagami, Yohei Kanzawa, Aiko Harada, Dai Aoki, Yoshiki Umezawa, Masataka Ono, So Sakamoto

**Affiliations:** ^1^ Department of General Medicine Yokohama City University School of Medicine Yokohama Japan; ^2^ Department of General Medicine Faculty of Medicine, Juntendo University Tokyo Japan; ^3^ Department of General Internal Medicine Kobe University Hospital Kobe Hyogo Japan; ^4^ Department of General Medicine Saiseikai Gotsu General Hospital Gotsu Shimane Japan; ^5^ Department of Emergency and General Medicine Ikeda City Hospital Ikeda Osaka Japan; ^6^ Department of General Medicine Dozen Hospital Tokyo Japan; ^7^ Department of General Internal Medicine Akashi Medical Center Akashi Hyogo Japan; ^8^ Department of Emergency and Critical Care Medicine Asahi General Hospital Chiba Japan

To the Editor,

Effective collaboration between specialist and primary care physicians is essential for improving patient outcomes and ensuring appropriate medical resource use.^1^, ^2^ However, their different perspectives can create barriers to care.^1^, ^2^ Referral of patients from primary care to specialist physicians can result in gaps in diagnostic workup and treatment, leading to unnecessary tests or delays.^2^, ^3^ Moreover, the lack of standards for consultation timing and information sharing contributes to misunderstandings. To address these issues, we—members of the Junior Doctors Association of the Japanese Society of Hospital General Medicine (JSHGM)—propose the “5G Approach,” (1. Gray Tolerance, 2. Guiding Criteria, 3. Gradual Transition, 4. Ground‐Level Coordination, and 5. Growth Through Follow‐up) five principles to promote effective collaboration between primary care and specialist physicians (Table 1). These principles were developed through a narrative literature review and discussions among eight primary care physicians with a median of 10 years' experience. The framework reflects clinical realities based on challenges from acute care settings and aligns with established concepts in the literature on collaborative care.^4^, ^5^ It has been informally applied in case discussions to improve communication between primary care and specialist physicians.

**TABLE 1:** The 5G approach.

	Principle	Definition	Practical example	Barriers	Enablers
1	Gray Tolerance	Accepting diagnostic uncertainty and sharing it transparently between primary care and specialist physicians	A primary care physician refers to a febrile patient with suspected pyelonephritis but acknowledges uncertainty regarding the focus of infection	Overemphasis on definitive diagnosis before referral	Communication of both confirmed and uncertain elements of the diagnosis
2	Guiding Criteria	Establishing shared criteria for consultation timing and patient transfer	For patients with altered mental status not clearly attributable to stroke, seizure, or metabolic disturbance, primary care physicians consult the neurology department based on preestablished criteria	Lack of predefined criteria	Institutional protocols and pre‐arranged referral conditions (e.g., sepsis with atypical presentation)
3	Gradual Transition	Facilitating stepwise handoff depending on timing, patient complexity, and resource availability	A patient with anaphylaxis is initially observed in the emergency department and then transitioned to care by a specialist physician after stabilization	Time constraints during shift changes and night hours	Flexibility in consultation timing, with daytime preference or use of observation periods
4	Ground‐Level Coordination	Promoting on‐site communication and coordination to align care goals	A specialist physician visits the emergency department to directly discuss patient disposition with the emergency team	Limited opportunity for face‐to‐face handover	Encouragement of bedside interaction or brief updates during busy hours
5	Growth Through Follow‐up	Engaging in mutual feedback and shared learning through postconsultation outcome review	The primary care physician follows up on the inpatient course of a patient that they referred and discusses the case with the specialist physician	Lack of feedback loop after consultation	Scheduled case reviews or informal feedback on shared patients

In primary care, treatment plans are often made before confirming the diagnosis.^4^ Primary care physicians must make decisions to mitigate risk while navigating uncertainty.^4^ Specialist physicians, in turn, are expected to understand and respond appropriately to this uncertainty.^1^, ^6^ However, discrepancies in perception may arise between the two regarding the degree of diagnostic certainty.^6^, ^7^ To prevent this, primary care physicians must acknowledge and share diagnostic uncertainty with specialist physicians,^6^ clearly communicating which aspects of the diagnosis are certain and which are uncertain, and specialist physicians should use this information to respond flexibly and manage time effectively.

To facilitate the transition from primary care to specialist care, the appropriate timing of consultations and the criteria for transfer need to be clarified.^1^, ^2^, ^7^ In practice, lack of clarity can result in missed opportunities for timely interventions.^1^, ^2^, ^7^ Thus, setting clear criteria for specialist consultation and patient transfer from primary to specialist care is necessary.^1^, ^2^, ^7^ Moreover, even when the diagnosis is uncertain, sharing transfer criteria can streamline care, promote appropriate medical resource use, and optimize patient outcomes.^1^, ^2^, ^7^

Depending on the patient's condition, gradual transfer of care from primary care to specialist care may be preferable.^7^ Flexibility is also essential in adapting approaches based on the time of day (daytime, nighttime, weekdays, and holidays),^2^, ^7^ and the availability of medical resources including testing equipment and medical staffing need to be considered, especially during nights and holidays, using predefined standards.

Increasing direct communication between primary care and specialist physicians and building mutual trust are important for enhancing collaboration.^1^, ^2^ Face‐to‐face sharing of patient information and discussion of treatment plans can help prevent misunderstandings and inconsistent patient management.^1^, ^2^

In primary care, reflection on treatment choice is often insufficient. However, appropriate feedback after consultations allows both primary care and specialist physicians to improve quality of care, enhance clinical judgment, and accumulate knowledge.^1^ Additionally, tracking patient outcomes and reviewing treatment appropriateness enables the application of insights to future care, promoting better decision‐making and improving the care process.^1^

Applying these five principles comprising the “5G Approach” could promote collaboration between primary care and specialist physicians and enhance the quality of patient care. Additional challenges in collaboration need to be identified and case studies of successful interventions need to be publicized.

## AUTHOR CONTRIBUTIONS

**Kosuke Ishizuka:** Writing – original draft; conceptualization; visualization. **Taiju Miyagami:** Writing – review and editing. **Yohei Kanzawa:** Writing – review and editing. **Aiko Harada:** Writing – review and editing. **Dai Aoki:** Writing – review and editing. **Yoshiki Umezawa:** Writing – review and editing. **Masataka Ono:** Writing – review and editing. **So Sakamoto:** Writing – review and editing; conceptualization; visualization; supervision.

## CONFLICT OF INTEREST STATEMENT

The authors state that they have no conflict of interest.
